# YAP/ACSL4 Pathway-Mediated Ferroptosis Promotes Renal Fibrosis in the Presence of Kidney Stones

**DOI:** 10.3390/biomedicines11102692

**Published:** 2023-10-01

**Authors:** Lei Li, Zehua Ye, Yuqi Xia, Bojun Li, Lijia Chen, Xinzhou Yan, Tianhui Yuan, Baofeng Song, Weimin Yu, Ting Rao, Fangyou Lin, Xiangjun Zhou, Fan Cheng

**Affiliations:** Department of Urology, Renmin Hospital of Wuhan University, Wuhan 430060, China; lilei981225@163.com (L.L.); yezehua0704@163.com (Z.Y.);

**Keywords:** calcium oxalate crystal, ferroptosis, kidney stone, renal fibrosis, YAP–ACSL4 axis

## Abstract

The potential association between calcium oxalate stones and renal fibrosis has been extensively investigated; however, the underlying mechanisms remain unclear. Ferroptosis is a novel form of cell death characterized by iron-dependent lipid peroxidation and regulated by acyl coenzyme A synthase long-chain family member 4 (ACSL4). Yes-associated protein (YAP), a transcriptional co-activator in the Hippo pathway, promotes ferroptosis by modulating ACSL4 expression. Nevertheless, the involvement of YAP–ACSL4 axis-mediated ferroptosis in calcium oxalate crystal deposition-induced renal fibrosis and its molecular mechanisms have not been elucidated. In this study, we investigated ACSL4 expression and ferroptosis activation in the kidney tissues of patients with calcium oxalate stones and in mice using single-cell sequencing, transcriptome RNA sequencing, immunohistochemical analysis, and Western blot analysis. In vivo and in vitro experiments demonstrated that inhibiting ferroptosis or ACSL4 mitigated calcium oxalate crystal-induced renal fibrosis. Furthermore, YAP expression was elevated in the kidney tissues of patients with calcium oxalate stones and in calcium oxalate crystal-stimulated human renal tubular epithelial cell lines. Mechanistically, in calcium oxalate crystal-stimulated human renal tubular epithelial cell lines, activated YAP translocated to the nucleus and enhanced ACSL4 expression, consequently inducing cellular ferroptosis. Moreover, YAP silencing suppressed ferroptosis by downregulating ACSL4 expression, thereby attenuating calcium oxalate crystal-induced renal fibrosis. Conclusively, our findings suggest that YAP–ACSL4-mediated ferroptosis represents an important mechanism underlying the induction of renal fibrosis by calcium oxalate crystal deposition. Targeting the YAP–ACSL4 axis and ferroptosis may therefore hold promise as a potential therapeutic approach for preventing renal fibrosis in patients with kidney stones.

## 1. Introduction

Nephrolithiasis is a common kidney disease influenced by genetic, dietary, and environmental factors, and its incidence and prevalence are on the rise [1]. Although minimally invasive surgery is an effective treatment for kidney stones, studies have indicated a high recurrence rate after surgery, resulting in a chronic condition that imposes a substantial societal and economic burden [2]. Kidney stones can be classified into different types based on mineral deposits, including calcium oxalate (CaOx), calcium phosphate, and uric acid stones. Among these, CaOx stones are the most prevalent [1,3]. CaOx has a tendency to supersaturate and form crystals that adhere to the epithelial surface of the renal tubules or the renal interstitium. The deposition of CaOx crystals can cause damage to the surrounding cells and tissues, triggering an inflammatory response that ultimately leads to chronic kidney disease and renal fibrosis [4,5]. Renal fibrosis is frequently observed in the histopathological examination of patients with kidney stones; nevertheless, the precise underlying mechanism of renal fibrosis induced by CaOx crystal remains elusive.

Ferroptosis is a distinct form of programmed cell death characterized by iron-dependent lipid peroxidation, setting it apart from apoptosis, necrosis, and autophagy [6]. The underlying mechanism of ferroptosis involves intracellular accumulation of free ferrous in the cell and subsequent oxidation of membrane phospholipids by a large quantity of free radicals via the Fenton reaction. This process leads to the buildup of lipid peroxides, ultimately resulting in cell death [7]. Ferroptosis is now recognized as being closely implicated in the pathophysiology of various diseases, including tumors, neurological disorders, ischemia–reperfusion injury, cardiovascular conditions, and renal ailments [8]. In a study by Wang et al., ferroptosis was found to be activated in a model of acute kidney injury, and inhibiting ferroptosis significantly mitigated renal ischemia–reperfusion as well as folic acid-induced acute kidney injury [9]. In our previous study, we demonstrated that inhibiting ferroptosis attenuated renal injury and renal fibrosis in mice with CaOx stones, possibly through its impact on epithelial–mesenchymal transition (EMT) [10]. Acyl-CoA synthetase long-chain family member 4 (ACSL4) is an enzyme that regulates lipid metabolism through its involvement in lipid peroxidation. ACSL4 converts fatty acids to fatty acid acyl coenzyme A esters [11]. Doll et al. identified ACSL4 as a crucial player in ferroptosis through CRISPR-based genetic screening and analysis of resistant cells. ACSL4 promotes ferroptosis by catalyzing the biosynthesis of lipids containing polyunsaturated fatty acids, leading to the accumulation of lipid peroxidation products and subsequent induction of ferroptosis [12]. ACSL4 has been implicated in various diseases such as tumors, ischemia–reperfusion injury, and acute renal injury [9,13,14]. Yes-associated protein (YAP) is a transcriptional co-activator protein in the Hippo signaling pathway that regulates cellular processes, such as cell growth, proliferation, differentiation, and migration, and also participates in antioxidation and apoptosis-related physiopathological processes [15]. Recent studies have shown that activated YAP upregulates the expression of ACSL4, consequently inducing ferroptosis [16,17,18,19,20] However, the role of YAP-mediated modulation of ACSL4-induced ferroptosis in CaOx crystal deposition-induced renal fibrosis remains unexplored, and the underlying mechanism remains unclear. Therefore, investigating the mechanism of action of ACSL4 and its upstream regulators provides a promising approach to target ACSL4 for the treatment of ferroptosis-related diseases.

In our present study, we developed a mouse model of CaOx kidney stones to investigate the effect of ACSL4 expression and ferroptosis activation on renal fibrosis. We employed an ACSL4-specific inhibitor and silenced ACSL4 gene expression to determine the impact on ferroptosis activation and consequently on renal injury and renal fibrosis, both in vivo and in vitro. To further understand the upstream regulatory mechanism of ACSL4, we aimed to identify its key upstream regulator. Our findings provide insights into the potential application of the YAP–ACSL4 pathway as a promising target for the prevention of renal fibrosis in patients with kidney stones.

## 2. Materials and Methods

### 2.1. Human Specimen

Kidney specimens were collected from six patients with non-functioning kidneys due to CaOx kidney stones (experimental group). Adjacent non-tumorous kidney tissues from six patients who underwent radical nephrectomy served as the control group. The specimens were obtained from the Department of Urology of the Renmin Hospital of Wuhan University and approved by the Ethics Committee of the Renmin Hospital of Wuhan University (Approval number: WDRY2021-KS047).

### 2.2. Animal Experiments

C57/BL6 mice weighing 24–28 g were acclimatized for one week before the establishment of the CaOx kidney stone model. Glyoxylic acid monohydrate (G10601, 120 mg/kg, Sigma-Aldrich, St. Louis, MO, USA) was administered daily via intraperitoneal injection for 6 or 12 days to induce CaOx kidney stones. To verify the effect of ferroptosis on renal injury and fibrosis caused by CaOx crystals, consecutive intraperitoneal injections of Ferrostatin-1 (Fer-1) (S7243, 10 mg/kg, Selleck, Houston, TX, USA), oral consumption of sterile water containing abemaciclib (LY2835219, 30 mg/kg, Selleck, Houston, TX, USA), and intraperitoneal injections of YAP inhibitor verteporfin (S1786, 10 mg/kg, Selleck, Houston, TX, USA) were administered for a duration of 12 days. The control group received daily intraperitoneal injections of the same volume of saline as the model group. All animal experiments were approved by the Animal Experimentation Welfare and Ethics Committee of Wuhan University Renmin Hospital (Approval number: WDRM-20200604).

### 2.3. Cell Culture and Transient Transfection

Human renal tubular epithelial cells (HK-2) obtained from the Cell Bank of the Chinese Academy of Sciences were cultured in DMEM/F12 medium supplemented with 10% fetal bovine serum (Gibco, Waltham, MA, USA) and 100 U/mL penicillin/streptomycin at an incubator at 37 °C with 5% CO_2_.

To construct the cell model, calcium oxalate monohydrate (COM) was added at various concentrations for 24 h to the cells upon reaching 80% confluence. Transfections of control small interfering RNA fragments (Con-siRNA), ACSL4-siRNA, YAP-siRNA, empty plasmid vector, YAP-plasmid (all from GenePharma, Shanghai, China), and Lipofectamine 2000 (Invitrogen, Waltham, MA, USA) were performed according to the manufacturer’s protocol. ACSL4-siRNA sequences were sense 5′-GAGGCUUCCUAUCUGAUUATT-3′ and antisense 5′-UAAUCAGAUAGGAAGCCUCTT-3′. YAP-siRNA sequences were sense 5′-CUGGUCAAAUACAGAUCAUTT-3′ and antisense 5′-AUGAUCUGUAUUUGACCAGTT-3′.

### 2.4. Transcriptome RNA Sequencing and Bioinformatics Analysis

Transcriptome sequencing was performed on three mice from the CaOx stone group and three control mice. Total RNA was extracted from the mouse kidney tissue using a TRIzol kit (Invitrogen Life Technologies, Carlsbad, CA, USA) and purified, fragmented, and reverse transcribed into cDNA to create a sequencing library.

Sequencing analysis was performed using BISEQ500 (BGI, Shenzhen, China). Differentially expressed genes were screened and subjected to volcano map, heat map, and GO analysis. *p* < 0.05 was considered statistically significant.

### 2.5. Quantitative Real-Time PCR (QPCR)

Total RNA was first extracted using TRIzol reagent (Invitrogen Life Technologies, Carlsbad, CA, USA), then reverse transcribed into cDNA using Takara RNA PCR kit (Takara Biotechnology, Otsu, Japan), and finally measured using En Turbo TM SYBR Green PCR Super Mix kit (Yeasen, Shanghai, China) on a Step One TM Real-Time PCR instrument to determine the expression levels of ACSL4 and YAP mRNA, and normalize them with GAPDH mRNA expression levels. GAPDH forward and reverse primers are 5′-GAACGTATCCCTGGACTAGG-3′ and 5′-TCAGACAGTGTAAGGGGTGAA-3′, respectively; ACSL4 forward and reverse primers are 5′-CATCCCTGGAGCAGATACTCT-3′ and 5′-TCACTTAGGATTTCCCTGGTCC-3′, respectively; YAP forward and reverse primers are 5′-ACCCTCGTTTTGCCATGAAC-3′ and 5′-TTGTTTCAACCGCAGTCTCTC-3′, respectively.

### 2.6. Determination of Biochemical Parameters

All mice were anaesthetized via intraperitoneal injection of 2% sodium pentobarbital (2.5 mL/kg), and peripheral blood was collected using a sterile syringe. Serum was obtained using centrifugation (3000 rpm with a radius of 13.5 cm at 4 °C for 15 min), and the levels of glutathione (GSH) (S0053, Beyotime Biotechnology, Shanghai, China), catalase (CAT) (BC0205, Solarbio, Beijing, China), and malondialdehyde (MDA) (S1031S, Beyotime Biotechnology, Shanghai, China) were measured using respective assay kits, according to the manufacturer’s protocol. OD values were measured using an enzyme marker, and serum urea nitrogen (BUN) and creatinine (Scr) levels were measured using fully automated biochemistry analytical instruments (DR-200Bs).

### 2.7. Histopathological Analysis

All mice were exposed to CO_2_ (30% CO_2_). They were cervically dislocated, and the kidneys were removed bilaterally. One side of the kidney was fixed in 4% paraformaldehyde and embedded in paraffin. The paraffin block was sectioned, and the sections were stained with hematoxylin and eosin (HE), Von Kossa, Masson, and Perls stains to assess renal tissue damage, CaOx crystals, fibrosis, and iron deposition. Scoring was based on the degree of tubular damage, ranging from 0–4: 0 = normal; 1 = mild damage (tubular damage < 25%); 2 = moderate (25% < tubular damage < 49%); 3 = severe (50% < tubular damage < 75%); 4 = extensive damage (tubular damage > 75%). ImageJ software, version 1.52a (NIH, Bethesda, USA) was used for the quantitative analysis of staining results.

### 2.8. Western Blot

Kidney tissues and HK-2 cells were placed on ice and lysed by adding RIPA lysis solution, PMSF and phosphatase inhibitor (all from Servicebio, Wuhan, China) for 30 min. After the protein concentration was determined by BCA method (Beyotime Biotechnology, Shanghai, China), 20 μg of protein was taken for SDS-PAGE electrophoresis, electrotransferred to PVDF membrane, put into TBST containing 0.1% Tween-20 and 5% skim milk for 1 h. The primary antibodies against these targets, YAP (14074, CST, Danvers, MA, USA), ACSL4 (ET7111-43, Huabio, Hangzhou, China), Collagen Ⅰ (ab34710, Abcam, Cambridge, UK), fibronectin (ab2413, Abcam, Cambridge, UK), α-SMA (GB111364, ServiceBio, Wuhan, China), P53 (ab26, Abcam, Cambridge, UK), GPX4 (ET1706-45, Huabio, Hangzhou, China), SLC7A11 (HA600098, Huabio, Hangzhou, China), GAPDH (GB15002, ServiceBio, Wuhan, China), Histone H3 (GB11102, ServiceBio, Wuhan, China), and incubated at 4 °C overnight. The secondary antibodies containing goat anti-mouse/rabbit IgG polymer (SA00001-1, SA0001-0, proteinTech, Wuhan, China) were added and incubated at 37 °C for 1 h. After washing with TBST, Chemi Doc imaging system (Bio-Rad, Hercules, CA, USA) was used to detect protein blots, and Image J software, version 1.52a (NIH, Bethesda, USA) for semi-quantitative analysis. 

### 2.9. Immunofluorescence

The 4 μm thick mouse kidney sections and HK-2 cell crawls were blocked with 5% bovine serum albumin (BSA) for 30 min, and then incubated with dilutions of primary antibodies YAP, ACSL4, GPX4, SLC7A11, and α-SMA at 4 °C overnight. Next, sections and crawls were incubated with pairs of applied fluorescently labeled secondary antibodies at 37 °C for 1 h, and the nuclei were double-stained with DAPI under light-protected conditions, and images were captured using fluorescence microscopy and analyzed with ImageJ software, version 1.52a (NIH, Bethesda, USA).

### 2.10. Transmission Electron Microscopy (TEM)

A total of 1 mm^3^ of fresh kidney cortex was removed, fixed in 2.5% glutaraldehyde and 1% osmium tetroxide, then dehydrated with gradient ethanol solvent and embedded in epoxy resin. The sections were sectioned using an ultrathin sectioning machine and then stained with 2% uranyl acetate saturated alcohol solution and lead citrate for 15 min, and the images were observed by transmission electron microscopy.

### 2.11. Cell Viability Assay

HK-2 cells were inoculated and cultured in 96-well plates, and after treatment with different concentrations of COM for 24 h, 10 μL of cell counting kit-8 (CCK-8, Beyotime Biotechnology, Shanghai, China) was added to each well and incubated at 37 °C for 1 h. The absorbance of each well was measured at 450 nm using an automated microplate reader, and the experiment was repeated three times for each group.

### 2.12. Lipid Peroxidation Assay

A C11 BODIPY581/591 lipid peroxidation fluorescent probe (MAOKANGBIO, MX5211-1 MG, Shanghai, China) was used to assess lipid peroxidation and antioxidation in HK-2 cells. Cells were incubated with C11 BODIPY581/591 for 30 min and then analyzed using confocal fluorescence microscopy. Fluorescence signals at 505–550 nm and >580 nm were detected with excitation from a 488–565 nm laser, respectively. Images capturing the relevant fluorescence signals were collected and quantified for lipid peroxidation levels based on the arithmetic mean ± SEM of the measured fluorescence signals. C11 BODIPY 581/591, a lipophilic dye, exhibits a green fluorescence when oxidized and red fluorescence when unoxidized.

Additionally, intracellular levels of reactive oxygen species (ROS) were measured using flow cytometry. The cells were incubated with a medium containing C11 BODIPY reagent, and subsequently, the PBS-washed cells were filtered through a cell filter. The ROS levels were measured using flow cytometry.

### 2.13. Immunohistochemistry

The 4-μm-thick kidney tissue sections were immersed in EDTA-buffered repair cassette for 1 min, incubated with 3% hydrogen peroxide solution at 37 °C for 25 min under light protection, washed three times with PBS, and blocked with 5% BSA for 30 min. The sections were then incubated with the ACSL4 primary antibody at 4 °C overnight and exposed to the enzyme-labeled goat anti-rabbit IgG polymer secondary antibody for 1 h the next day. Hematoxylin was used to re-stain the nuclei. After dehydration, the sections were sealed and observed by microscope. Ten areas were randomly selected and evaluated the percentage of positive cells and then ImageJ software, version 1.52a (NIH, Bethesda, USA) was used for semi-quantitative analysis.

### 2.14. Statistical Analysis

All experimental data were presented as mean ± SD. Statistical analysis was performed using GraphPad Prism, version 7.0 (GraphPad Software, San Diego, CA, USA). All experiments were performed in triplicate, and the results were averaged. Continuous variables were analyzed using one-way analysis of variance (ANOVA) and *t*-test, whereas categorical variables were analyzed using the chi-squared test. Statistical significance was defined as *p* < 0.05.

## 3. Results

### 3.1. Ferroptosis Is Activated in Mice with CaOx Kidney Stones

**Figure 1:**
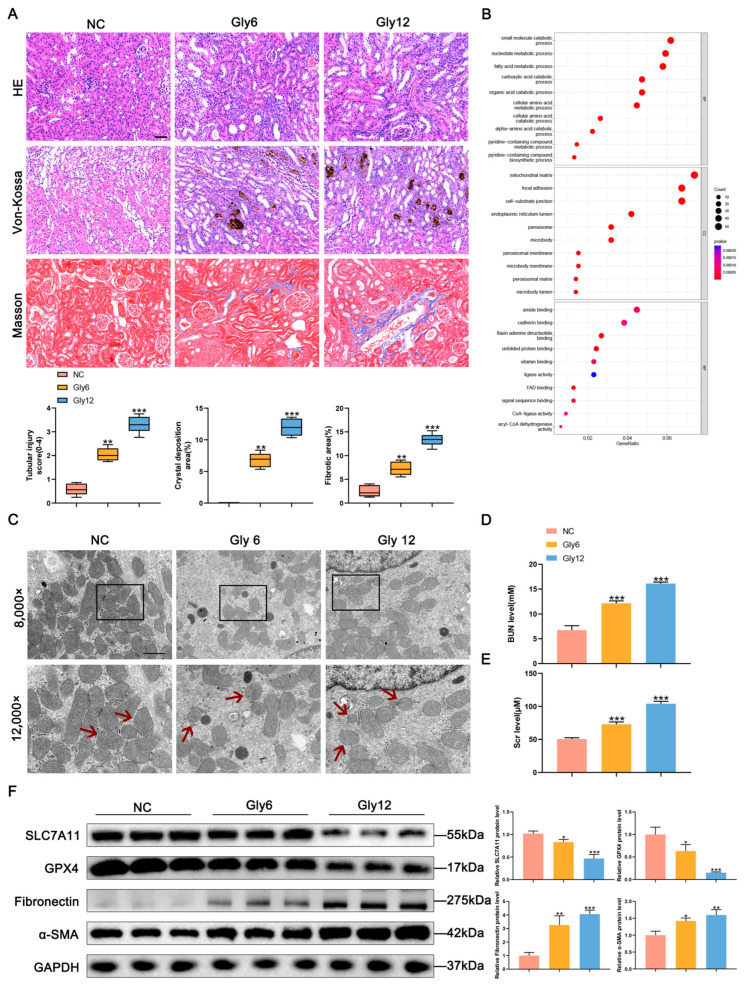
Ferroptosis is activated in mice with CaOx kidney stones. (**A**) HE, Von Kossa and Masson staining results to evaluate tubular damage, CaOx crystals and collagen fibrillation deposition (*n* = 5). The scale bar represents 50 µm. (**B**) GO enrichment analysis. (**C**) TEM showed shrunken mitochondria with outer membrane ruptured (indicated by red arrows) in mice with CaOx kidney stones. The scale bar represents 1 µm. (**D**,**E**) BUN and Scr levels of kidney tissue (*n* = 5). (**F**) Western blot analysis showed the expressions of SLC7A11, GPX4, fibronectin, and α-SMA in kidney tissues and quantification by densitometry. Data were presented as mean ± SD; * *p* < 0.05, ** *p* < 0.01, *** *p* < 0.001 vs. control group.

To assess the extent of ferroptosis and renal fibrosis in mice with CaOx kidney stones, we established CaOx kidney stone models by administering glyoxylic acid monohydrate via intraperitoneal injection for 6 and 12 consecutive days. HE staining revealed inflammatory cell infiltration and disruption of renal tubular structures following glyoxylic acid monohydrate stimulation (Figure 1A). Von Kossa and Masson staining indicated increased collagen fibrillation associated with the deposition of CaOx crystals in renal tissue, showing a time-dependent increase. Transcriptome RNA sequencing identified 5775 differentially expressed genes, including 4364 upregulated genes and 1411 downregulated genes, in the kidneys of CaOx kidney stone mice and control mice (Figure S1A). GO enrichment analysis showed a significant activation of the fatty acid pathway associated with ferroptosis in mice with CaOx kidney stones (Figure 1B). TEM images demonstrated altered mitochondrial structure in mice with CaOx kidney stones, with intracellular mitochondria shrinking and becoming smaller, reduced, or absent mitochondrial cristae, increased membrane density, and increased mitochondrial damage with time (Figure 1C). Measurement of serum BUN and Scr levels revealed the most severe kidney damage in mice after 12 days (Figure 1D,E). Western blot analysis showed decreased expression of SLC7A11 and GPX4, which are key proteins in the regulation of ferroptosis, and increased expression of the fibrosis-related proteins fibronectin and α-SMA in mice with CaOx kidney stones, with a more pronounced effect over time (Figure 1F). These findings indicate the activation of ferroptosis in mice with CaOx kidney stones, implicating the involvement of ferroptosis in the fibrotic process following renal injury caused by CaOx crystals.

### 3.2. Inhibition of Ferroptosis Alleviates Renal Fibrosis

**Figure 2:**
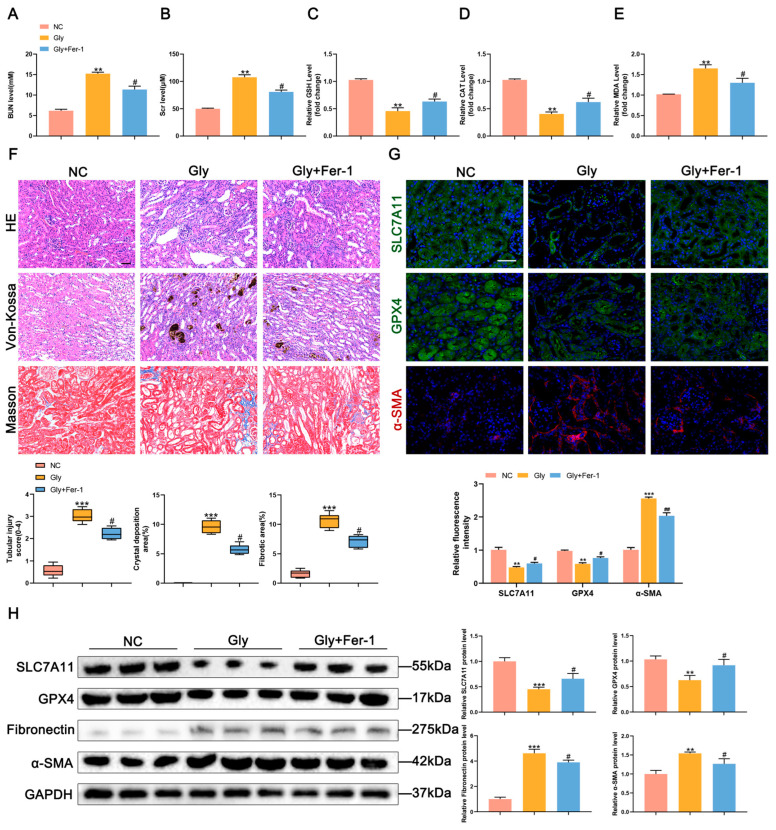
Inhibition of ferroptosis alleviates renal fibrosis. (**A**–**E**) Measurement of the levels of BUN, Scr, GSH, CAT, and MDA in the serum of mice (*n* = 5). (**F**) HE, Von Kossa and Masson staining results to evaluate tubular damage, CaOx crystals and collagen fibrillation deposition (*n* = 5). The scale bar represents 50 µm. (**G**) Immunofluorescence staining analysis of SLC7A11, GPX4 and α-SMA expressions in mouse kidney tissues and semi-quantitative analysis (*n* = 5). The scale bar represents 50 µm. (**H**) Western blot analysis showed the expressions of SLC7A11, GPX4, fibronectin and α-SMA in kidney tissues and quantification by densitometry. Data were presented as mean ± SD; ** *p* < 0.01, *** *p* < 0.001 vs. control group; # *p* < 0.05, ## *p* < 0.01 vs. Gly group.

To further elucidate the impact of ferroptosis on fibrosis following CaOx crystal-induced kidney injury, we employed the ferroptosis inhibitor Fer-1 to suppress ferroptosis in mice. Treatment with Fer-1 resulted in a significant decrease in serum BUN, Scr, and MDA, as well as a significant increase in reduced GSH and CAT levels in mice in the Gly + Fer-1 group compared to those in the Gly group (Figure 2A–E). Histological examination through HE, Masson, and Von Kossa staining demonstrated that Fer-1 treatment mitigated kidney damage, collagen fibril deposition, and CaOx crystal accumulation in mice in the Gly + Fer-1 group compared to those in the Gly group (Figure 2F). Immunohistochemical fluorescence and Western blot analysis further confirmed that Fer-1 treatment upregulated the expression of the ferroptosis-regulating proteins SLC7A11 and GPX4 in mice in the treatment group compared to those in the Gly group. Conversely, the expression of the fibrosis-related proteins fibronectin and α-SMA in the treatment group was downregulated compared with that in the Gly group (Figure 2G,H). These findings indicate that Fer-1 inhibits ferroptosis and attenuates fibrosis in mice with CaOx kidney stones.

### 3.3. ACSL4 Expression Is Upregulated in CaOx Kidney Stones

**Figure 3:**
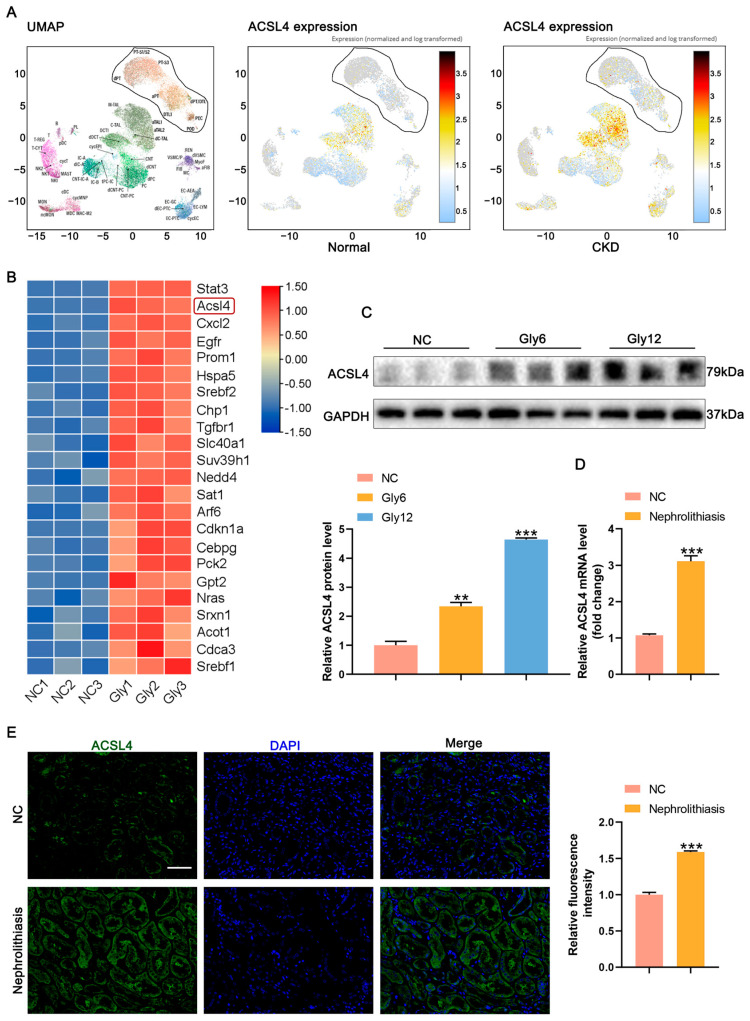
ACSL4 expression is upregulated in CaOx kidney stones. (**A**) Single-cell sequencing database of ACSL4 expression in normal subjects and patients with CKD. (**B**) RNA sequencing analysis of the transcriptomes of control and Gly group mice, red represents highly expressed genes and blue represents low expressed genes (*n* = 3). (**C**) Western blot analysis showed the expressions of ACSL4 expression in kidney tissues and quantification by densitometry (*n* = 5). (**D**,**E**) qPCR and immunofluorescence analysis of ACSL4 expression in the kidney of normal and kidney stone patients and semi-quantitative analysis (*n* = 6). The scale bar represents 50 µm. Data were presented as mean ± SD; ** *p* < 0.01, *** *p* < 0.001 vs. control group.

Comparison of single-cell sequencing data from the KPMP website between normal subjects and patients with CKD revealed upregulated ACSL4 expression in the proximal renal tubular epithelial cells of patients with CKD (Figure 3A). Transcriptome RNA sequencing analysis identified differentially expressed genes (Figure S1A), and a heat map specifically highlighting ferroptosis-related genes showed increased expression, with ACSL4 exhibiting particularly elevated levels (Figure 3B). Western blot analysis confirmed the increased expression of ACSL4 in mice with CaOx kidney stones, which showed a significant increase over time (Figure 3C). Immunohistochemical analysis provided consistent results with the Western blot findings (Figure S1B). Furthermore, qPCR and fluorescence immunohistochemistry results demonstrated increased ACSL4 expression in the kidney tissues derived from patients with kidney stones than in normal kidney tissue (Figure 3D,E).

### 3.4. ACSL4-Induced Ferroptosis Promotes Renal Fibrosis in Mice with CaOx Kidney Stones

**Figure 4:**
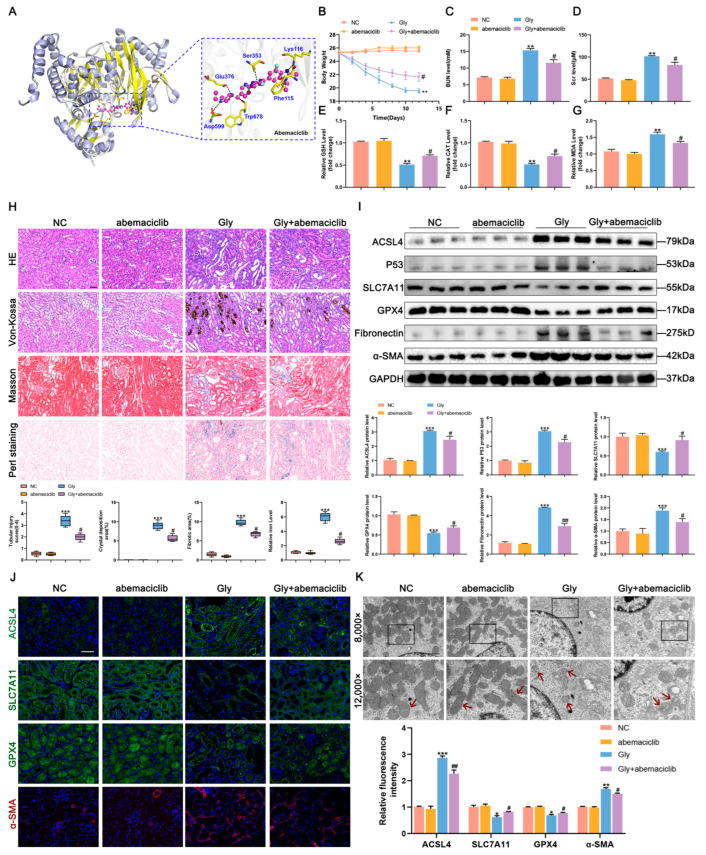
ACSL4-induced ferroptosis promotes renal fibrosis in mice with CaOx kidney stones. (**A**) Overall docking plot of abemaciclib and ACSL4. (**B**) Body weight changes in mice (*n* = 5). (**C**–**G**) Measurement of the levels of BUN, Scr, GSH, CAT, and MDA in the serum of mice (*n* = 5). (**H**) HE, Von Kossa, Masson, and Perls staining to assess tubular damage, CaOx crystals, collagen fibrillation and iron deposition (*n* = 5). The scale bar represents 50 µm. (**I**) Western blot analysis showed the expressions of ACSL4, P53, SLC7A11, GPX4, fibronectin, and α-SMA in mouse kidney tissues and quantification by densitometry. (**J**) Immunofluorescence staining analysis of ACSL4, SLC7A11, GPX4, and α-SMA expressions in mouse kidney tissues and semi-quantitative analysis (*n* = 5). The scale bar represents 50 µm. (**K**) TEM revealed mitochondrial damage (indicated by red arrows) in mice with CaOx kidney stones. The scale bar represents 1 µm. Data were presented as mean ± SD; * *p* < 0.05, ** *p* < 0.01, *** *p* < 0.001 vs. control group; # *p* < 0.05, ## *p* < 0.01 vs. Gly group.

ACSL4, a member of the acyl-CoA synthetases (ACS) family, plays a role in lipid peroxidation and acts as one of the upstream regulators of ferroptosis. Previous studies have shown that abemaciclib can effectively and selectively inhibit ACSL4 activity [21]. By aligning the predicted protein structure of ACSL4 from the AlphaFold website with abemaciclib using Moe software, version 2022.02, it was identified that abemaciclib can target ACSL4 (Figure 4A). Intraperitoneal administration of abemaciclib to mice treated with glyoxylic acid monohydrate significantly alleviated weight loss; reduced the serum levels of BUN, Scr and MDA; and increased GSH and CAT levels compared to those in the Gly group (Figure 4B–G). Histological analysis through HE, Von Kossa, Masson, and Perls staining confirmed reduced tubular damage, CaOx crystals, collagen fibril deposition, and iron deposition in mice treated with both glyoxylic acid monohydrate stimulation and abemaciclib compared to the those in the Gly group (Figure 4H). Compared with mice in the control and abemaciclib groups, those in the Gly group exhibited increased expression of ACSL4, the positive ferroptosis regulator P53, and the fibrosis-related proteins fibronectin and α-SMA, along with decreased expression of the negative ferroptosis regulator SLC7A11 and GPX4. In contrast, treatment of mice in the Gly group with abemaciclib significantly reversed the elevated levels of P53, fibronectin and α-SMA, while increasing the expression of SLC7A11 and GPX4. (Figure 4I,J). Furthermore, TEM observation showed that the Gly group mice treated with abemaciclib displayed less mitochondrial damage compared to the Gly mice not treated with amebaciclib (Figure 4K).

**Figure 5:**
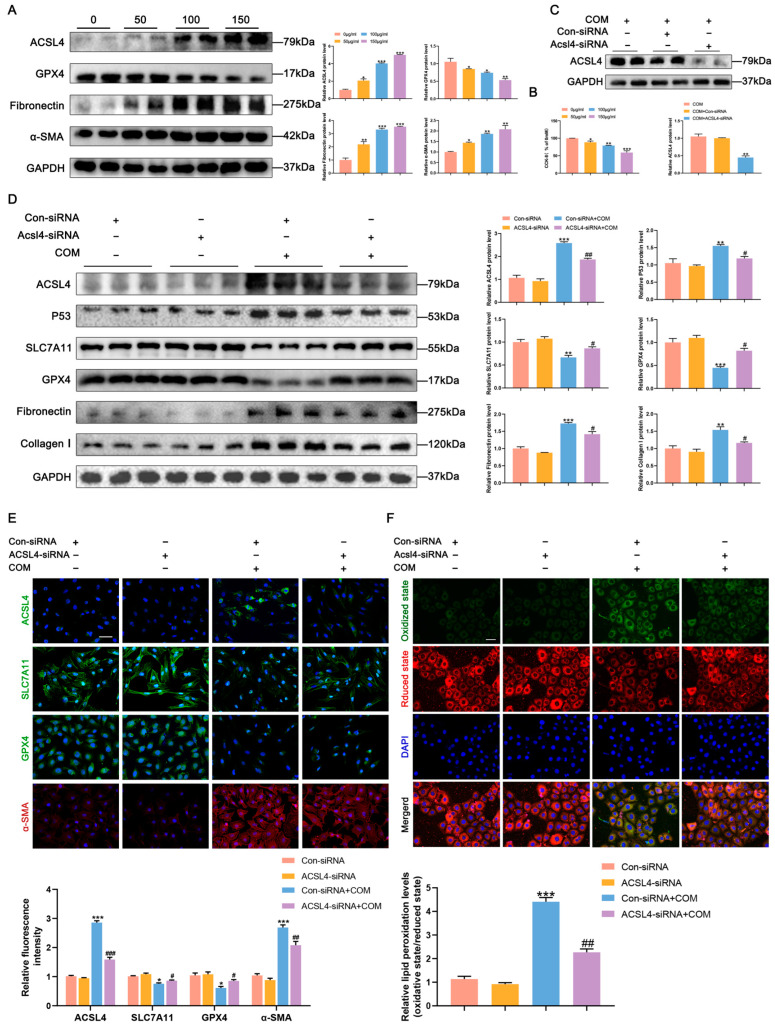
ACSL4-induced ferroptosis promotes COM-induced fibrosis in HK-2 cells. (**A**) Western blot analysis showed the expressions of ACSL4, GPX4, fibronectin and α-SMA after stimulation of HK-2 cells with different concentrations of COM and quantification by densitometry. (**B**) Cell viability of HK-2 under different concentrations of COM stimulation. * *p* < 0.05, ** *p* < 0.01, *** *p* < 0.001 vs. control group. (**C**) Western blots validated ACSL4-siRNA knockdown efficiency. ** *p* < 0.01 vs. COM group. (**D**) Western blot analysis showed the expressions of P53, SLC7A11, GPX4, fibronectin and Collagen I after knockdown of ACSL4 and quantification by densitometry. (**E**) Immunofluorescence analysis of SLC7A11, GPX4 and α-SMA expressions in HK-2 cells after knockdown of ACSL4 and semi-quantitative analysis. The scale bar represents 50 µm. (**F**) Measurement of lipid peroxidation in HK-2 cells by the C11 BODIPY581/591 fluorescent probe. The scale bar represents 50 µm. * *p* < 0.05, ** *p* < 0.01, *** *p* < 0.001 vs. Con-siRNA group; # *p* < 0.05, ## *p* < 0.01, ### *p* < 0.001 vs. Con-siRNA + COM group. Data were presented as mean ± SD.

To further validate the results obtained with animal model, we established an in vitro model by stimulating HK-2 cells with COM. Increasing concentrations of COM stimulation resulted in upregulated expression of ACSL4 and the fibrosis-related proteins fibronectin and α-SMA, along with downregulated expression of the ferroptosis-negative regulatory protein GPX4 in a COM concentration-dependent manner (Figure 5A). The optimal concentration of COM determined through a CCK-8 viability assay was found to be 100 μg/mL (Figure 5B). After confirming the knockdown efficiency of ACSL4-siRNA through Western blot validation, ACSL4-siRNA was transfected into each group of HK-2 cells, resulting in the inhibition of ferroptosis and fibrosis in the COM group (Figure 5C,D). Immunofluorescence results were consistent with these findings (Figure 5E). Additionally, cells were stained using the C11 BODIPY581/591 lipid peroxidation fluorescent probe, with green and red fluorescence indicating oxidized and unoxidized states, respectively. The results demonstrated significant lipid peroxidation in HK-2 cells following COM stimulation, and knockdown of ACSL4 reduced the levels of lipid peroxidation (Figure 5F). These findings collectively indicate that inhibition of ACSL4 suppresses CaOx-induced lipid peroxidation, thereby mitigating ferroptosis and renal fibrosis.

### 3.5. YAP Is Involved in Regulating the Expression of ACSL4 Transcript Levels, and Inhibition of YAP Inhibits ACSL4-Mediated Ferroptosis and Reverses Renal Fibrosis

**Figure 6:**
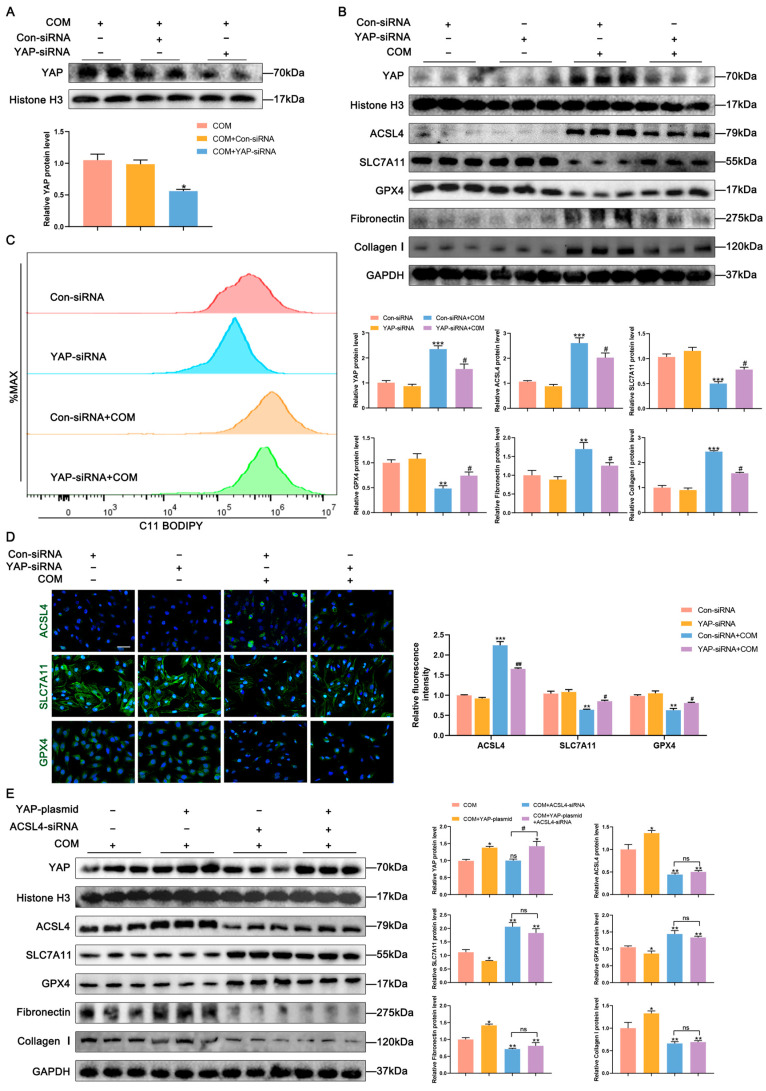
YAP is involved in regulating the expression of ACSL4 transcript levels, and inhibition of YAP inhibits ACSL4-mediated ferroptosis and reverses renal fibrosis. (**A**) Western blots validated YAP-siRNA knockdown efficiency. * *p* < 0.05 vs. COM group.(**B**) Western blot analysis showed the expressions of ACSL4, SLC7A11, GPX4, fibronectin, and Collagen I after knockdown of YAP and quantification by densitometry. (**C**) Measurement of changes in intracellular ROS in HK-2 cells after knockdown of YAP by flow cytometry. (**D**) Immunofluorescence analysis of ACSL4, SLC7A11, and GPX4 expressions in HK-2 cells following knockdown of YAP and semi-quantitative analysis. The scale bar represents 50 µm. * *p* < 0.05, ** *p* < 0.01, *** *p* < 0.001 vs. Con-siRNA group; # *p* < 0.05, ## *p* < 0.01 vs. Con-siRNA + COM group. (**E**) Western blot analysis showed the expressions of SLC7A11, GPX4, fibronectin, and Collagen I expressions after overexpression of YAP, with or without ACSL4-siRNA and quantification by densitometry. * *p* < 0.05, ** *p* < 0.01 vs. COM group; # *p* < 0.05 vs. ACSL4-siRNA + COM group; ns, no statistical significance. Data were presented as mean ± SD.

Previous studies have demonstrated the ability of YAP to act as a transcriptional co-activator on the ACSL4 promoter [17]. Fluorescence co-localization analysis of COM-treated HK-2 cells revealed predominantly nuclear localization of YAP and predominantly cytoplasmic localization of ACSL4, with increased expression of both proteins following COM stimulation (Figure S1C). This finding was further confirmed using Western blot analysis (Figure S1D). Additionally, qPCR analysis showed elevated YAP mRNA levels in the kidney tissues derived from patients with kidney stones compared to those in normal kidneys (Figure S1E). To investigate the role of YAP in ACSL4-mediated ferroptosis, YAP-siRNA was utilized in HK-2 cells, and efficient knockdown of YAP was confirmed using Western blot analysis (Figure 6A). Notably, Western blot and immunofluorescence results demonstrated that YAP knockdown in the COM group resulted in decreased intracellular ACSL4 expression, along with reduced ferroptosis and fibrosis, compared to that in the COM group (Figure 6B,D). Flow cytometry revealed that intracellular ROS increased in HK-2 cells under COM stimulation but decreased after YAP knockdown (Figure 6C). The findings obtained with si-YAP were subsequently corroborated through in vivo experiments using the YAP inhibitor verteporfin (Figure S2). Moreover, Western blot analysis showed that YAP overexpression resulted in increased expression of ACSL4, fibronectin, and collagen I, and decreased expression of SLC7A11 and GPX4. Conversely, the changes in ferroptosis and fibrosis-related proteins were not statistically significant in the YAP overexpression + ACSL4 knockdown group compared to those in the ACSL4 knockdown group alone, indicating that YAP activates ferroptosis and fibrosis by promoting ACSL4 expression (Figure 6E).

**Figure 7:**
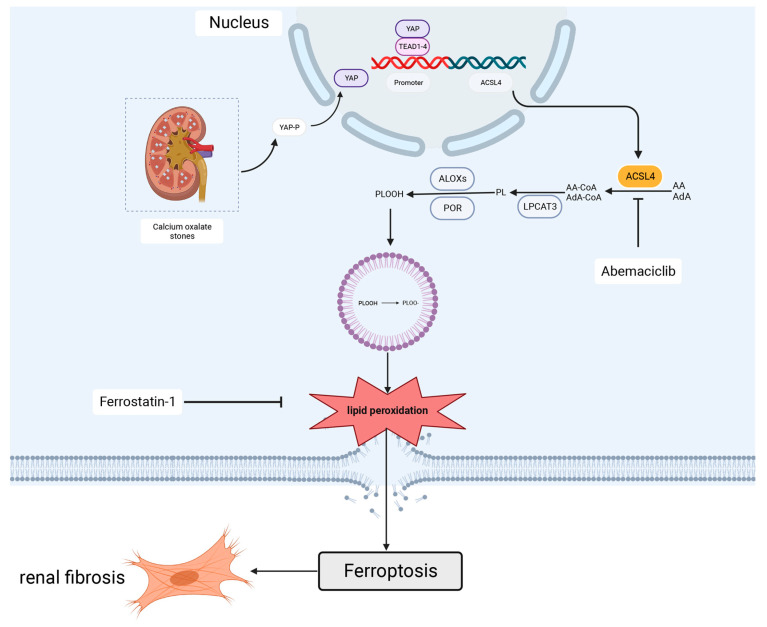
Inhibition of YAP mediates the downregulation of ACSL4 expression which, in turn, inhibits CaOx crystal-induced ferroptosis and renal fibrosis.

In conclusion, these results suggest that inhibition of YAP mediates the downregulation of ACSL4 expression, which in turn inhibits CaOx crystal-induced ferroptosis and renal fibrosis (Figure 7).

## 4. Discussion

Kidney stones, particularly CaOx stones, are prevalent mineral deposits found in the renal calyces and pelvis, either as free particles or attached to the renal papillae [3]. The attachment of CaOx crystals to the renal tubular epithelium leads to cellular damage, pro-inflammatory mediator release, cell death, leukocyte infiltration, and ultimately culminates in tubular atrophy and renal fibrosis [22,23]. Additionally, ureteral obstruction caused by stone detachment can induce tubular and interstitial damage, contributing further to the development of renal fibrosis [24]. Hu et al. demonstrated that in a mouse model of CaOx kidney stones, crystal deposition induced epithelial–mesenchymal transition (EMT) and renal fibrosis in renal tubular epithelial cells. Moreover, inhibiting stone formation with pharmacological interventions reduced crystal deposition-induced renal fibrosis [25]. Clinical studies have also revealed significantly elevated levels of renal fibrosis in kidney tissue samples from patients with kidney stones, indicating a strong association between kidney stones and renal fibrosis [26].

It is widely recognized that ferroptosis represents an adaptive and programmed form of cell death. Induction of ferroptosis in cancer cells has been shown to inhibit tumor growth, while inhibition of ferroptosis has been implicated in various diseases such as ischemia–reperfusion injury, ischemic cardiomyopathy, neurodegenerative diseases, organ transplantation, renal injury, and renal fibrosis, where it can inhibit disease progression [7]. Consistent with our previous study, our present findings demonstrate significantly elevated levels of ferroptosis-related proteins and renal fibrosis-related markers in renal tissues from CaOx stone mice [10]. To investigate the relationship between ferroptosis and CaOx crystal-induced kidney injury and renal fibrosis, we employed Fer-1, a potent and selective inhibitor of ferroptosis. Fer-1 exerts an inhibitory effect on ferroptosis mainly by suppressing lipid peroxidation and reducing the production of lipid hydroperoxides [27]. Our findings provide further support for the involvement of ferroptosis in the pathogenesis of CaOx crystal deposition-induced renal injury and renal fibrosis in a mouse model of CaOx stones. Moreover, our results suggest that inhibition of ferroptosis may hold clinical potential for preventing CaOx stone-induced renal injury and renal fibrosis.

ACSL4 not only serves as a marker for the occurrence of ferroptosis in cells, but also plays a crucial role as a ferroptosis regulator in the pathogenesis of various diseases, contributing to the induction of cellular ferroptosis [9,13,28,29,30,31]. Studies conducted in a mouse model of ischemia–reperfusion injury have shown upregulation of ACSL4 in ischemic tissues, and inhibiting ACSL4 has been found to significantly attenuate tissue and organ damage by inhibiting ferroptosis [9,13,31]. Furthermore, increased expression of ACSL4 has been observed in various tumor tissues, particularly in triple-negative breast cancer, and tumor cells with elevated ACSL4 expression may exhibit greater sensitivity to radiotherapy and chemotherapy [30]. A previous study demonstrated that COM induced ferroptosis in HK-2 cells in vitro and significantly upregulated the expression of ACSL4 [32]. In our study, we investigated the expression of ACSL4 in the kidney tissues of mice with CaOx stone using transcriptomic and single-cell sequencing analysis. The results revealed that ACSL4 expression was significantly upregulated in the kidney tissues of CaOx stone mice. Furthermore, our data demonstrated a similar upregulation of ACSL4 expression in kidney tissues from patients with CaOx stones, further suggesting that ACSL4 is closely associated with CaOx stone-induced kidney injury and renal fibrosis. Previous research has indicated that thiazolidinediones, a class of hypoglycemic drugs, can effectively inhibit ferroptosis by suppressing ACSL4 expression. However, their clinical utility is limited due to various side effects, including fluid retention and weight gain [17]. Abemaciclib is a novel ACSL4-specific inhibitor that has been shown to exhibit a stronger affinity for ACSL4 than thiazolidinediones [21]. Previous studies have demonstrated the efficacy of abemaciclib in attenuating liver injury and liver fibrosis in a mouse model of nonalcoholic fatty liver disease by selectively inhibiting ACSL4 expression [21]. In our study, pharmacological inhibition of ACSL4 using abemaciclib effectively blocked the development of renal fibrosis following kidney injury in vivo. In addition, in vitro experiments further revealed that genetic inhibition of ACSL4 was able to suppress COM-induced ferroptosis and fibrosis levels in HK-2 cells. Thus, our data suggest that ACSL4-induced ferroptosis plays a facilitative role in the development of CaOx crystal deposition-induced kidney injury and renal fibrosis. Moreover, our study highlights that abemaciclib has therapeutic potential as a selective inhibitor of ACSL4.

The YAP protein, a core effector downstream of the Hippo signaling pathway, plays a crucial role in intracellular signaling and gene transcription regulation as an intracellular linker and transcriptional co-activator, and has also been implicated in various fibrotic diseases [33]. Previous studies have shown that YAP is significantly upregulated in various tumor tissues and that activation of YAP can induce ferroptosis in tumor cells, including malignant mesothelioma cells [34]. However, in certain tumors, YAP inhibition leads to ferroptosis induction, such as in sorafenib-resistant hepatocellular carcinoma [35]. Therefore, the role of YAP in ferroptosis regulation appears to be cell type- and context-dependent. He et al. reported that in a mouse model of exertional heat stroke (EHS)-induced rhabdomyolysis (RM), YAP expression was significantly enhanced in the skeletal muscle tissues, and that mechanistically, YAP was able to induce ferroptosis in the skeletal muscle cells by upregulating ACSL4 expression, which eventually led to RM [17]. In our current study, YAP expression was significantly upregulated in the kidney tissues of patients and mice with CaOx stones. Given the known influence of YAP on ACSL4 expression, and the significant expression of ACSL4 in the kidney tissues of patients and mice with CaOx stones, we further investigated the interplay between YAP and ACSL4. Cytofluorimetric localization revealed that YAP was primarily localized in the nucleus, whereas ACSL4 was primarily localized in the cytoplasm. Furthermore, we found through in vitro and in vivo experiments that inhibiting YAP expression significantly reduces ACSL4 levels and alleviates CaOx-induced ferroptosis and fibrosis. Conversely, overexpression of YAP upregulated the expression of ACSL4 and promoted COM-induced ferroptosis and fibrosis. Importantly, we further found that overexpression of YAP failed to promote COM-induced ferroptosis and fibrosis by knocking down the ACSL4 gene in HK-2 cells. As such, these findings suggest that YAP exacerbates renal fibrosis induced by CaOx crystal deposition by inducing cellular ferroptosis through upregulation of ACSL4 expression. However, our study has certain limitations. YAP functions as a transcriptional co-activator, and its transcriptional activity is dependent on interactions with transcription factors such as TEAD1-4. In the EHS RM model, YAP has been shown to regulate the transcription and expression of ACSL4 through interactions with TEAD1/TEAD4 [17]. Similarly, TAZ, another core downstream effector of the Hippo signaling pathway, is associated with various pro-fibrotic signaling pathways, such as the TGF-β signaling pathway [33]. Therefore, YAP may also be involved in CaOx crystal deposition-induced renal fibrosis through other pathways, which we intend to investigate in future studies.

## 5. Conclusions

In summary, our study suggests that YAP induces cellular ferroptosis and promotes renal fibrosis induced by CaOx crystal through upregulation of ACSL4 expression. Targeting ferroptosis and the YAP–ACSL4 axis may represent a potential therapeutic strategy for preventing renal fibrosis associated with CaOx crystal deposition.

## Data Availability

The datasets in this study are available from the corresponding author upon reasonable request.

## Supplementary Materials

The following supporting information can be downloaded at: https://www.mdpi.com/article/10.3390/biomedicines11102692/s1, Figure S1: Expression of ACSL4 and YAP in vivo and in vitro CaOx kidney stone models and patients; Figure S2: Pharmacologic inhibition of YAP by verteporfin and its effect on CaOx kidney stone-induced injury.
