# Supplementary material for: YAP/ACSL4 Pathway-Mediated Ferroptosis Promotes Renal Fibrosis in the Presence of Kidney Stones
Source: Biomedicines. 2023 Oct 1;11(10):2692. doi: 10.3390/biomedicines11102692 (PMC10603838; doi:10.3390/biomedicines11102692)
Supplement: Supplementary file 1 [file biomedicines-11-02692-s001.zip › biomedicines-2569929 supplementary.pdf]

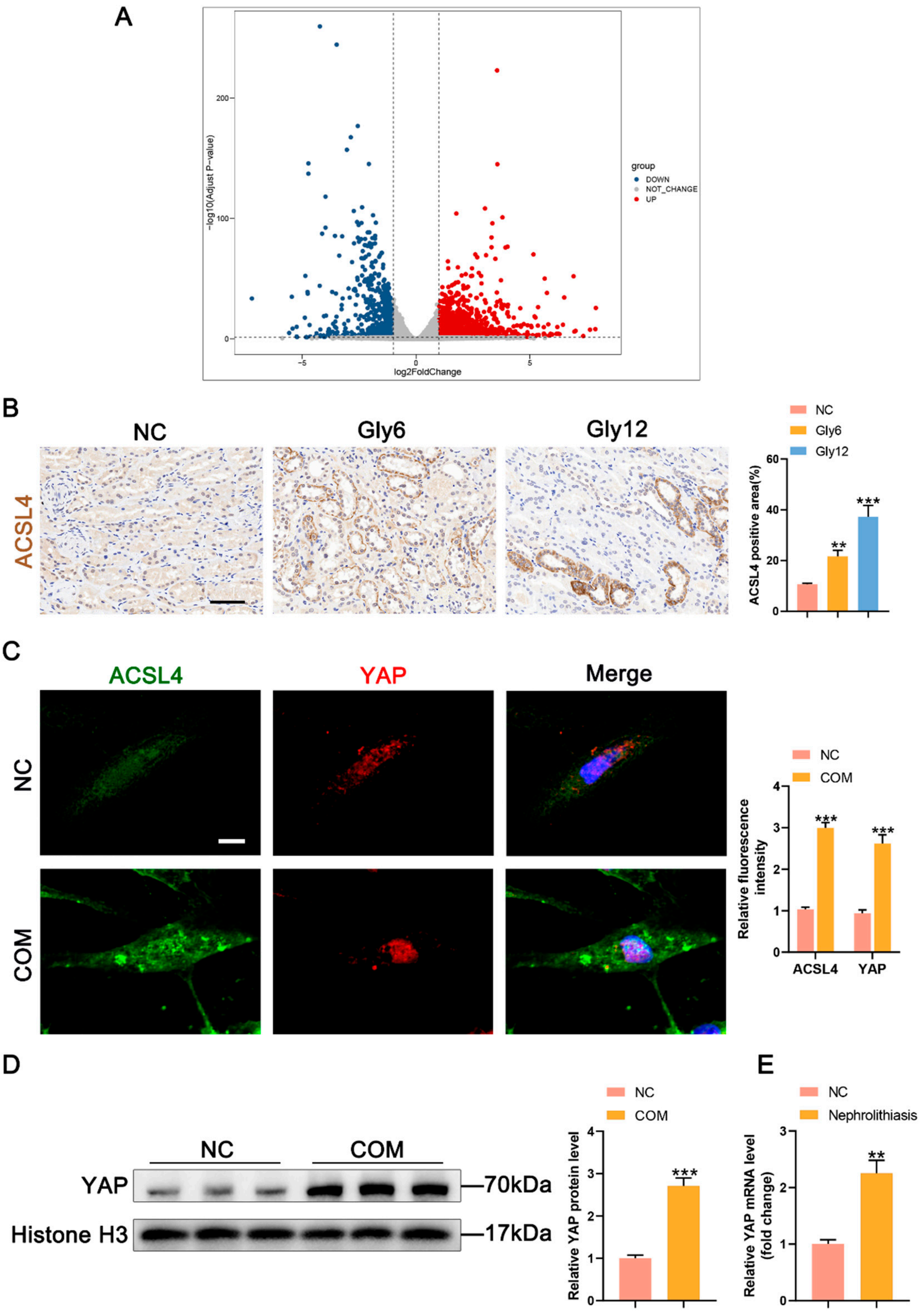

Supplementary Figure S1. Expression of ACSL4 and YAP in vivo and in vitro CaOx kidney stone models and patients. (A) RNA-seq volcano plot of normal and CaOx kidney stone mice (*n*=3). (B) Immunohistochemical analysis of ACSL4 expression over time in mice with CaOx kidney stones and semi-quantitative analysis (*n*=5). The scale bar represents 50μm. $^{**}P < 0.01$, $^{***}P < 0.001$ vs. control group. (C-D) The location and expression of ACSL4 and YAP in cells were analyzed by immunofluorescence, while the content of YAP was analyzed by western blots and quantification by densitometry. The scale

bar represents 10μm. ***$P < 0.001$ vs. control group. (E) qPCR analysis of YAP expression in the kidney of normal and kidney stone patients and semi-quantitative analysis (n=6). **$P < 0.01$vs. control group. Data were presented as mean ± SD.

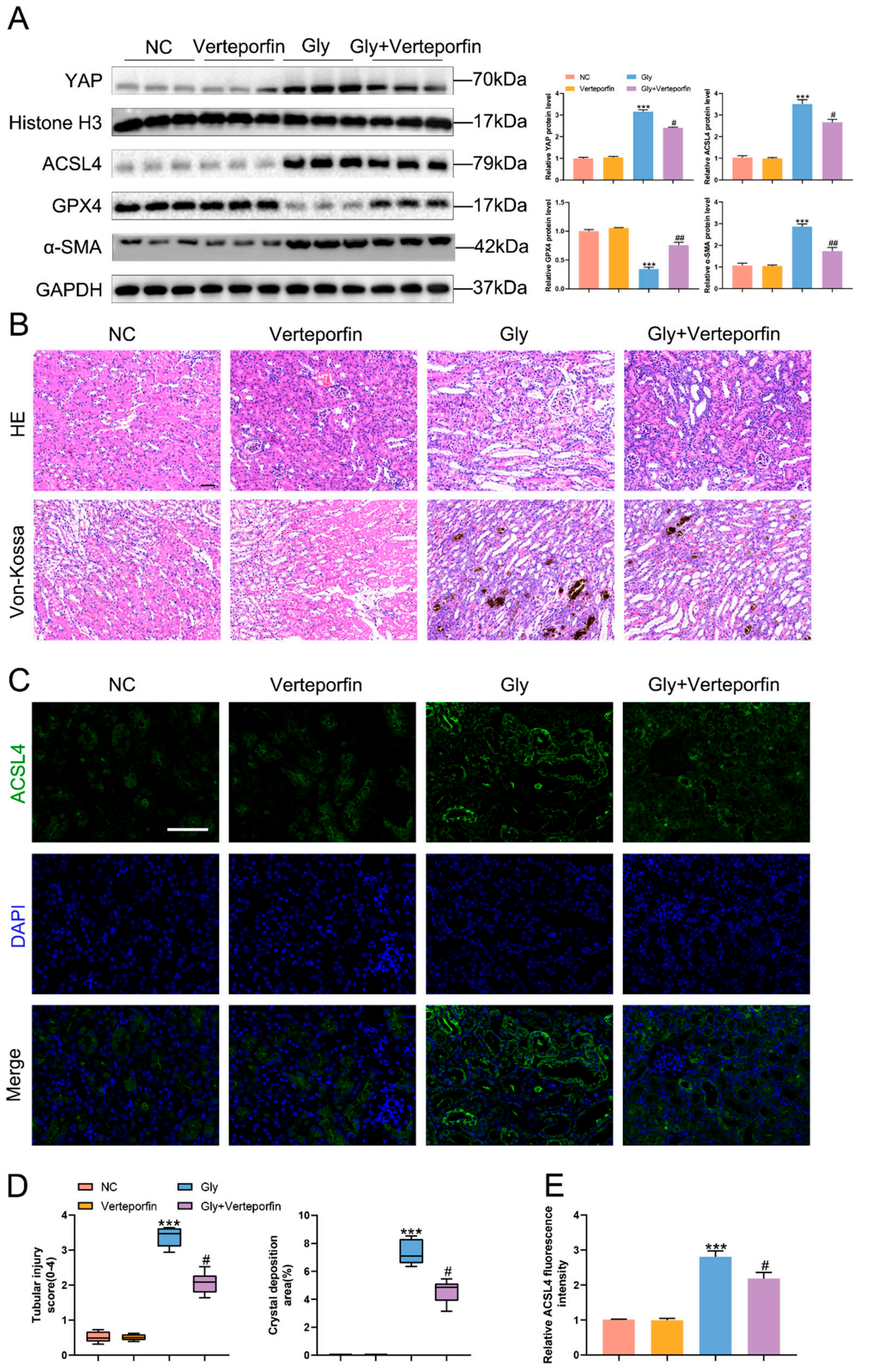

Supplementary Figure S2. Pharmacologic inhibition of YAP by verteporfin and its effect on CaOx kidney stone-induced injury. (A) Western blot analysis showed the expressions of YAP, ACSL4, GPX4 and α-SMA in mouse kidney tissues and quantification by densitometry. (B, D) HE and Von-Kossa staining to assess tubular damage and CaOx crystal deposition (*n*=*5*). The scale bar represents 50 μm. (C, E) Immunofluorescence staining analysis of ACSL4 expression in mouse kidney tissues and semi-quantitative analysis (*n*=*5*). The scale bar represents 50 μm. Data were presented as mean ± SD; ****P < 0.001* vs. control group; *#P<0.05, ##P<0.01* vs. Gly group.
